# Effect of Azilsartan on clinical blood pressure reduction compared to other angiotensin receptor blockers: a systematic review and meta-analysis

**DOI:** 10.1097/MS9.0000000000001547

**Published:** 2023-12-08

**Authors:** Qaisar Ali Khan, Shalini Sharma, Ittehad ul Mulk, David Li, Naod F. Belay, Muhammad Afzal, Ameer Mustafa Farrukh, Muhammad Asad, Abdul Baqi, Bader Semakieh

**Affiliations:** a Khyber Teaching Hospital, MTI KTH; b Lady Reading Hospital, Peshawar, Pakistan; c Texas A&M School of Medicine, Bryan, TX; d Idaho College of Osteopathic Medicine, Meridian, ID; e Michigan State University, East Lansing, MI; f St. George’s University School of Medicine, True Blue, Grenada; g University of Galway School of Medicine, Galway, Ireland; h Mercy Saint Vincent Medical Center, Toledo, OH; i Arkansas College of Osteopathic Medicine, Fort Smith, AR

**Keywords:** angiotensin receptor blocker, azilsartan, blood pressure reduction, hypertensive patients

## Abstract

**Background:**

Hypertension has significantly contributed to morbidity and mortality, necessitating effective management. Angiotensin receptor blockers (ARBs) have emerged as a cornerstone in hypertension treatment. Azilsartan, a relatively recent addition to the ARB family, offers unique characteristics, including prodrug activation. This systematic review and meta-analysis aimed to evaluate Azilsartan’s role in reducing clinical blood pressure compared to other ARBs and determine the most effective dosage.

**Methods:**

Following PRISMA guidelines, a comprehensive literature search was conducted in Medline, Web of Science, Cochrane Library, and clinicaltrials.gov. Eligible studies included adult hypertensive patients receiving Azilsartan compared to other ARBs, with clinical systolic blood pressure (SBP) and diastolic blood pressure (DBP) outcomes. Data extraction and quality assessment were performed, and statistical analysis employed comprehensive meta-analysis (CMA) software.

**Results:**

Eleven randomized controlled trials encompassing 18 studies involving 6024 patients were included. Azilsartan demonstrated significant reductions in clinical SBP (mean difference=−2.85 mmHg) and DBP (mean difference=−2.095 mmHg) compared to other ARBs. Higher doses of Azilsartan showed greater efficacy, with 80 mg exhibiting the most substantial reduction in SBP. The analysis emphasized the need for more studies investigating lower Azilsartan doses (10 and 20 mg).

**Conclusion:**

This systematic review and meta-analysis underscore Azilsartan’s effectiveness in reducing SBP and DBP. Dose-dependent effects emphasize the importance of optimal dosing when prescribing Azilsartan. These findings provide valuable insights for clinicians in managing hypertension effectively and call for further research, primarily focusing on lower Azilsartan doses and a more diverse patient population.

## Introduction

Highlights

- Compared to other angiotensin receptor blockers, Azilsartan is statistically significant in reducing the systolic blood pressure and diastolic blood pressure of hypertensive patients.
- The dose-dependent effects of Azilsartan highlight the importance of considering the optimal dosing regimens when prescribing to hypertensive patients.
- The findings of this research will prove valuable to clinicians for their everyday practice in managing hypertension effectively.

Essential hypertension, currently defined as the systolic blood pressure (SBP) of equal to or more than 130 mmHg and diastolic blood pressure (DBP) of more than 80 mm Hg, has proved to be one of the most investigated problems in the previous century provided its link with multiple diseases including myocardial infarction, renal failure, and stroke^1^. Hence, it also stands as a significant contributor to morbidity and mortality. It is estimated that every year, at least 10 million people succumb to this preventable disease alone, with even more due to its fatal consequences^2^.

Multiple treatment and management options have been devised to deal with HTN. Among the various classes of antihypertensive medications available, angiotensin receptor blockers (ARBs) have emerged as a cornerstone and one of the first-line antihypertensive to be prescribed for managing hypertension^3^.

ARB’s mechanism of action can be understood by dissecting the renin-angiotensin activating system (RAAS). Renin is secreted by the kidney’s juxtaglomerular cells and catalyzes the conversion of angiotensinogen to angiotensin I (ATI) in the liver. Angiotensin-converting enzyme (ACE) and other non-ACE mechanisms convert ATI to angiotensin II (ATII). The main vasoactive peptide in the RAAS is ATII, which activates two receptors, AT1 and AT2. Increased blood pressure, systemic vascular resistance, sympathetic activity, sodium (Na), and water retention due to enhanced Na reabsorption in the proximal convoluted tubule are all effects of ATII activation of AT1 receptors. ARBs antagonize the effect of AII on AT1 receptors^4,5^. Common adverse effects associated with ARBs include dizziness, headache, and gastrointestinal disturbances^3^. Rarely, they may lead to more severe adverse events such as hyperkalemia, kidney dysfunction, and angioedema. Many drugs are approved by the United States Food and Drug Administration (FDA), including Candesartan, Eprosartan, Irbesartan, Losartan, Olmesartan, Telmisartan, and Valsartan. Azilsartan, a relatively recent addition to the ARB family, was approved in 2011^6^. Like other ARBs, Azilsartan works on the same mechanism, however, its effects are dose related. Studies have shown that repeating doses of Azilsartan medoxomil increases the plasma concentrations of angiotensin I, as well as angiotensin II, alongside Renin activity also increased, and at the same time decreases plasma aldosterone levels^7^. It has a unique prodrug feature that implies it is initially delivered in an inactive form and then metabolically converted in the body to its active form, Azilsartan medoxomil. When compared to other ARBs, this prodrug characteristic provides for better absorption and a longer duration of action^8^. Another factor that works in favour of Azilsartan is its safety profile. Azilsartan has been demonstrated to be well-tolerated with the most common adverse event presenting after its usage being diarrhoea. Other adverse events reported are hypotension, orthostatic hypotension, asthenia, nausea, fatigue, dizziness, muscle spasm, and cough. The laboratory parameters do not differ significantly as well among groups and includes slight rise in creatine at maximum dose (80 mg), that have been attributed to decrease in BP and low haematocrit^7^.

While multiple studies have assessed and compared Azilsartan with different hypertensive drugs, there needs to be more cohesive information and comparison of the drug with other ARBs. And the most effective dose of the drug. This systematic review and meta-analysis aim to critically evaluate Azilsartan’s role in reducing blood pressure compared to other ARBs and analyze the best dose, providing valuable insights into its clinical utility and safety profile for hypertensive patients.

## Materials and methods

Our present meta-analysis was pre-registered on PROSPERO and performed according to guidelines of Preferred Reporting Items for Systematic Reviews (PRISMA) guidelines^9^. The work has been reported in line with AMSTAR (Assessing the methodological quality of systematic reviews) guidelines.

### Literature search

A thorough search on Medline (via PubMed), Web of Science, Cochrane Library, and clinicaltrials.gov was performed to identify relevant articles from inception to August 2023. Bibliographies of the identified studies were also searched for other relevant articles. The following search terms were employed:

“Azilsartan”, “azilsartan medoxomil”, “Angiotensin Receptor Blockers”, “ARBs,” “Hypertension”, “High Blood Pressure,” “Blood Pressure Reduction”, “Blood Pressure Control”, “Angiotensin II Receptor Antagonists”

### Eligibility criteria

The eligibility criteria followed the PICOS strategy:

Population: Adult patients with Hypertension and taking any form of ARBs.

Intervention: Azilsartan.

Comparators: Other drugs of the same class (ARBs).

Study design: Randomized controlled trials (RCT) and observational studies comparing both drugs.

Outcome: Studies that reported clinical SBP and DBP between the groups after intervention.

Our exclusion criteria were as follow:

Non-English Language Studies: Studies published in languages other than English were excluded.

Non-Human Studies: Studies conducted on animals or in vitro experiments were excluded, as our primary interest was adult patients with hypertension.

Studies with Incomplete Data: Studies that lacked essential data on clinical blood pressure measurements.

Non-comparative studies: Studies that did not have a comparative design were excluded, as our aim was to assess the effectiveness of azilsartan compared to other ARBs.

Duplicate publications: Duplicate publications of the same study were removed.

### Study selection and data extraction

All titles and abstracts were screened for inclusion according to the abovementioned criteria. Full texts of selected articles were screened for in-depth review by two investigators, and data were extracted from eligible articles into a pre-structured Microsoft Excel data sheet (Version 2019, Microsoft). Disagreements were resolved by consultation with another author. The following data were extracted from the studies: First Author, the year of publication, country of origin, study design, sample size, age, sex, dosage of the drugs, follow-up time, presence of Diabetes and dyslipidemia, and finally, outcomes of interest.

### Quality assessment

The quality of our included studies was evaluated using the Cochrane risk of bias (RoB2) tool^10^, as all included studies were RCTs. Two independent reviewers performed the quality assessment, and any discrepancy was resolved by consultation with another author.

### Statistical analysis

Data analysis was performed using CMA version 3.0. Dichotomous data are presented as odds ratios (ORs) and continuous data as mean differences (MDs). Authors were emailed in case of missing data. A random-effects model was used to deal with the heterogeneity of included studies. An I^2^ index greater than 75% is demonstrated as high heterogeneity. A *P* value of less than 0.05 was considered statistically significant in all analyses.

## Results

### Literature search

**Figure 1:**
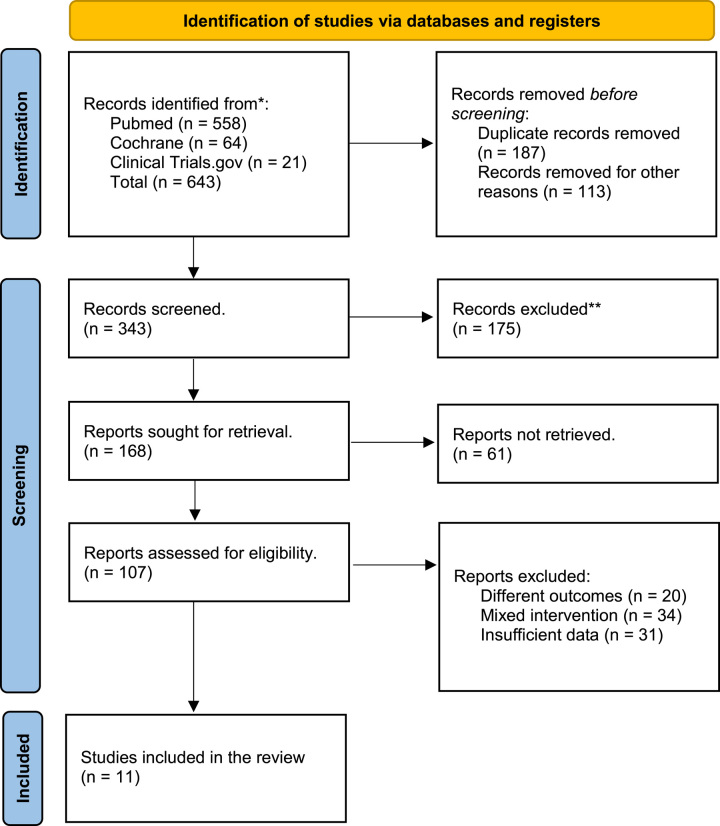
Preferred Reporting Items for Systematic Reviews and Meta-Analyses (PRISMA) flowchart of literature search.

A total of 643 articles were retrieved. After removing duplicates, 456 articles were screened via titles and abstracts. Ultimately, 107 articles were selected for in-depth review. Finally, 11 RCTs^11–21^, reporting data for 18 different regimens, were included in the final qualitative and quantitative meta-analysis. This selection process is illustrated in the PRISMA flowchart (Fig. 1).

### Study characteristics

The characteristics of the included studies are shown in Table 1. There were 3590 patients in the Azilsartan group and 2434 in the control group; hence a total of 6024 patients are included in this meta-analysis. The included studies were conducted in 4 countries. Of these studies, two had Olmesartan as a control^11,12^, three studies reported Valsartan^12,13,21^ and Candesartan^14,16,19^ as control, while four studies had Telmisartan as control^15,17,18,20^.

**Table 1:** Study characteristics

			Participants’ characteristics							
			Azilsartan		Control					
References	Study type	Country	*N* Age M/F	Dose (mg)	Drug *N* Age M/F	Dose (mg)	Total duration (weeks)	Diabetes	Dyslipidemia	Outcomes
Bakris *et al.* ^11^	RCT	USA	282 57.1 133/150	20	Olmesartan 282 58.9 140/142	40	6	N/A	N/A	- Clinical SBP and DBP
			283	40						
			285	80						
White *et al.* ^12^	RCT	USA	280 57±12 53%/48%	40190	Valsartan 282 55±11 54%/46%	320	6	N/A	N/A	- Clinical SBP and DBP - Clinical SBP and DBP
			285 56±11 53%/47%	80	Olmesartan 290 56±11 55%/45%	40				
Rakugi *et al.* ^14^	RCT	Japan	313 57.0 (± 9.69) 184/129	20 (8 weeks) → 40 (8 weeks)	Candesartan 309 56.9 (± 10.00)	8 (8 weeks) → 12 (8 weeks) 196/113	16	I= 61 C= 73	I= 182 C= 168	- Clinical SBP and DBP
Sica *et al.* ^13^	RCT	USA	327 57.8±12.1 164/163	40	Valsartan 309 58.1±10.9 176/152	320	24	N/A	N/A	- Clinical SBP and DBP
			329 56.8±10.7 169/160	80						
Meher *et al.* ^17^	RCT	India	24 54.42 ± 7.83 14/10	40	Telmisartan 23 53.09 ± 8.02 13/10	40	12	N/A	N/A	- Clinical SBP and DBP
Ito *et al.* ^16^	RCT	Japan	82	20	Candesartan 92	8	48	N/A	N/A	- Clinical SBP and DBP
Sinha *et al.* ^18^	RCT	India	98 47±10 53/45	40	Telmisartan 102 47 ± 10 47/55	40	6	N/A	N/A	- Clinical SBP and DBP
			103 49±10 45/58	80						
Garg *et al.* ^20^	RCT	India	350 50.56±14.98 196/154	40 → 80 (2 weeks	Telmisartan 350 49.64 ± 13.56 203/147	40 → 80 (2 weeks	12	N/A	N/A	- Clinical SBP and DBP
Wu *et al.* ^21^	RCT	China	190 57.4±9.5 107/92	40	Valsartan 204 56.80 ± 9.5 130/74	160	8	AZL (40): 14 (7%) AZL (80): 20 (9.6%) VAL: 14 (6.9%)	N/A	- Clinical SBP and DBP
			209 57.00±9.9 115/94	80						
Narusi *et al.* ^15^	RCT	Japan	17 63.2±12.76 7/10	20	Telmisartan 16 65.3 ± 9.107/9	40	12	33	N/A	- Clinical SBP and DBP
Takahara *et al.* ^19^	RCT	Japan	133 68±11 77/56	10	Candesartan 175 66±12 10372	8	16	AZL= 75 (56%) CAN= 108 (61%)	AZL= 70 (53%) CAN= 94 (53%)	- Clinical SBP and DBP

DBP, diastolic blood pressure; F, female; M, male; N/A, not available; RCT, randomized controlled trial; SBP, systolic blood pressure.

### Risk of bias assessment

According to the RoB2 tool, all our studies were low risk for Random sequencing and selective reporting. For Allocation concealment, White *et al.* ^12^ were marked high risk, while for blinding of outcome assessor, five of our studies were marked high risk^11,13,14,18,20^. For incomplete data, all studies had low risk of bias while for other sources, only one was marked unclear^12^. The quality assessment of the eleven RCTs is tabulated in detail in Table 2.

**Table 2:** Risk of bias of included studies.

Author, year	Random sequence generation	Allocation concealment	Selective reporting	Blinding of participants/personnel	Blinding of outcome assessment	Incomplete outcome data	Other sources of bias
Bakris *et al.*, 2011^11^	Low risk	Low risk	Low risk	Low risk	High risk	Low risk	Low risk
White *et al.*, 2011^12^	Low risk	High risk	Low risk	Low risk	Unclear	Low risk	Unclear
Rakugi *et al.*, 2011^14^	Low risk	Low risk	Low risk	Low risk	High risk	Low risk	Low risk
Sica *et al.*, 2011^13^	Low risk	Low risk	Low risk	Low risk	High risk	Low risk	Low risk
Meher *et al.*, 2022^17^	Low risk	Low risk	Low risk	Low risk	Unclear	Low risk	Low risk
Ito *et al.*, 2023^16^	Low risk	Low risk	Low risk	Low risk	Low risk	Low risk	Low risk
Sinha *et al.*, 2021^18^	Low risk	Low risk	Low risk	Low risk	High risk	Low risk	Low risk
Garg *et al.*, 2020^20^	Low risk	Low risk	Low risk	Low risk	High risk	Low risk	Low risk
Wu *et al.*, 2020^21^	Low risk	Low risk	Low risk	Low risk	High risk	Low risk	Low risk
Narusi *et al.*, 2019^15^	Low risk	Low risk	Low risk	Low risk	High risk	Low risk	Low risk
Takahara *et al.*, 2014^19^	Low risk	Low risk	Low risk	Low risk	High risk	Low risk	Low risk

### Publication bias

**Figure 2:**
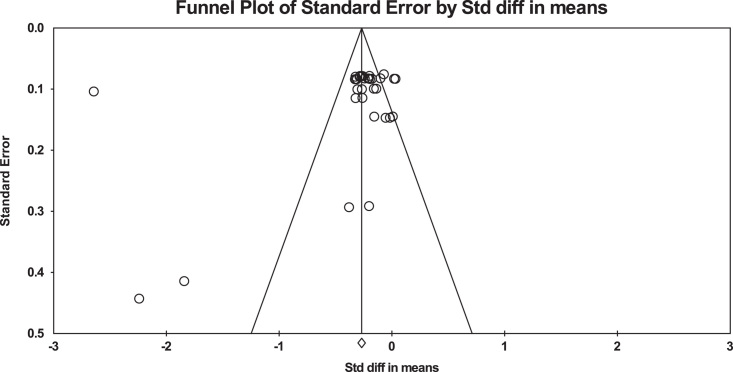
Publication bias.

The publication bias between studies is illustrated in Fig. 2.

### Quantitative analysis

#### Clinical SBP

All our studies reported clinical SBP as an outcome^11–21^. Our analysis found that Azilsartan reduced the SBP more than other ARBs. The analysis was statistically significant between the groups MD= −2.853 [95% CI= −3.807 (−2.240), *P*<0.001]. Moderate heterogeneity was found between studies in this analysis (I^2^ = 56%; *P* = 0.00).

**Figure 3:**
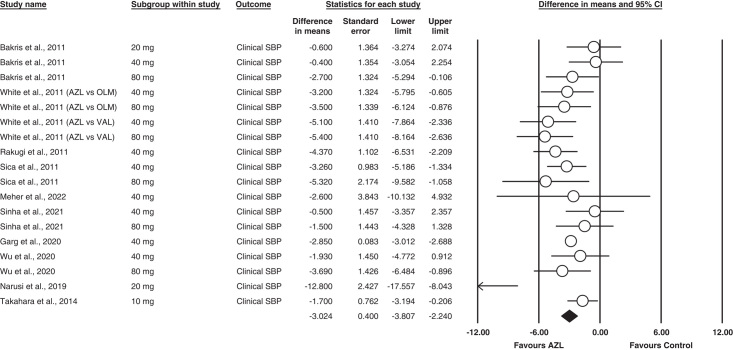
Forest plot for clinical systolic blood pressure.

The outcome is illustrated in Fig. 3.

#### Clinical DBP

Fifteen of the included eighteen studies reported clinical DBP as an outcome^12–14,17–21^. Our analysis found Azilsartan statistically significant in reducing the DBP compared to other ARBs MD= −2.095 [95% CI= −2.975 to (−1.215), *P*<0.001]. Significant heterogeneity was found between studies in this analysis (I^2^ = 81%; *P* = 0.001).

**Figure 4:**
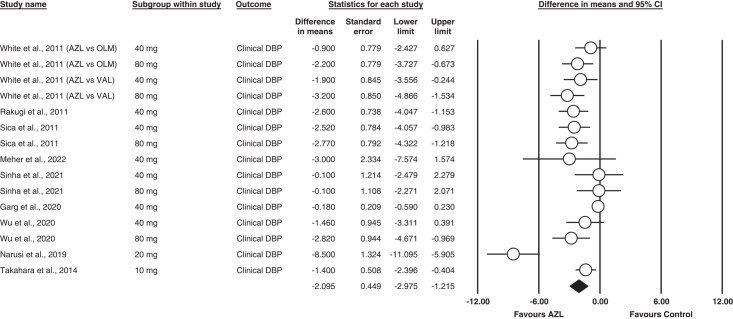
Forest plot for clinical diastolic blood pressure.

The outcome is illustrated in Fig. 4.

#### Subgroup analysis

We performed a subgroup analysis of our data based on doses of Azilsartan and comparative drugs used in our included studies.

##### 10 mg and 20 mg Azilsartan

Only one study reported our outcome of interest with 10 mg, favoring Azilsartan^19^. While only two studies^11,16^ reported Azilsartan in 20 mg doses, hence they were not analyzed separately.

##### 40 mg Azilsartan (SBP)

**Figure 5:**
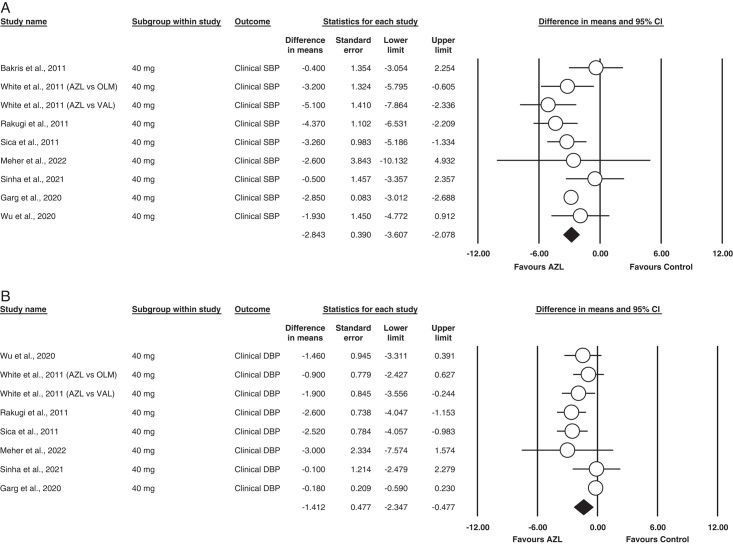
(A) Subgroup analysis of 40 mg AZL (clinical systolic blood pressure). (B) Subgroup analysis of 40 mg AZL (Clinical diastolic blood pressure).

Nine studies used 40 mg of Azilsartan as an intervention drug for clinical SPB^11–14,17,18,20,21^. The analysis revealed that the dose was statistically significant compared to other class drugs. MD= −2.843 [95% CI= −3.607 to (2.078), *P*<0.001]. Minimum heterogeneity was found between studies in this analysis (I^2^ = 27.0%; *P* = 0.203). The outcome is illustrated in Figure 5(A).

##### 40 mg Azilsartan (DBP)

Eight of the eighteen studies reported clinical DBP for 40 mg Azilsartan as an outcome^12–14,17,18,20,21^. Our analysis found Azilsartan statistically significant in reducing the DBP compared to other ARBs MD=−1.412 [95% CI= −2.347 to (0.471), *P*=0.003]. Applying the fixed model assumption, heterogeneity was calculated, which was borderline high in the outcome (I^2^ = 68%; *P* = 003).

The outcome is illustrated in Figure 5(B).

##### 80 mg Azilsartan (SBP)

Six studies utilized an 80 mg dose of Azilsartan for their studies^11–13^. When analyzed, it was statistically superior to the control of respected studies in bringing down clinical SBP in the cohort MD= −3.506 (95% CI= −4.674 to (−2.388), *P*<0.001]. No heterogeneity was found in between the studies (I^2^=0, *P*= 0.438).

**Figure 6:**
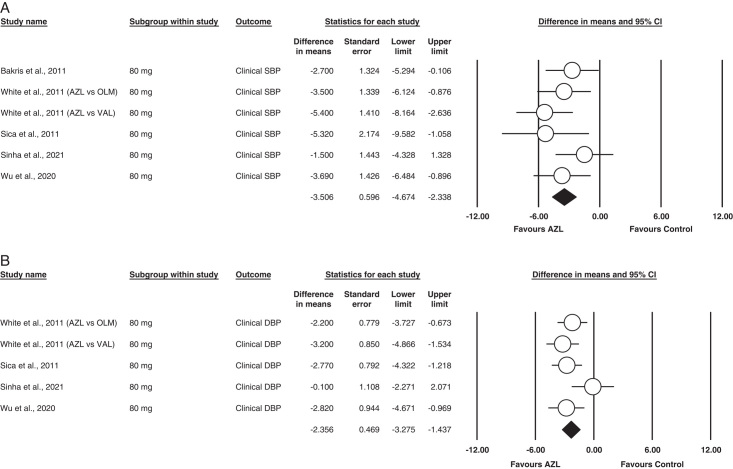
(A) Subgroup analysis of 80 mg AZL (Clinical systolic blood pressure). (B) Subgroup analysis of 80 mg AZL (clinical diastolic blood pressure).

The outcome is illustrated in Figure 6(A).

##### 80 mg Azilsartan (DBP)

Five studies utilized an 80-mg dose of Azilsartan for clinical DBP reporting^12,13,18,21^. Our analysis found it statistically superior to other ARBs MD= =−2.356 [95% CI= −3.257 to (−1.437), *P*<0.001]. Mild heterogeneity was found between the studies on applying the fixed model (I^2^=29%, *P*= 0.224).

The outcome is illustrated in Figure 6(B).

## Discussion

The findings of this systematic review and meta-analysis shed significant light on the clinical effectiveness of Azilsartan in managing hypertension, particularly concerning its impact on clinical SBP and DBP.

Our analysis revealed a substantial reduction in SBP (mean reduction=−2.85 mmHg) and DBP (mean reduction=−2.095 mmHg) among patients treated with Azilsartan, highlighting its efficacy as an antihypertensive medication. This is consistent with previously reported studies that proved its efficacy. However, these studies also had diuretics as a control, or the sample size was limited to a specific geographical location^22–25^. In another analysis, azilsartan medoxomil 80 mg topped with a 99% chance of being the best in class for systolic blood pressure reduction, followed by azilsartan medoxomil 40 mg, and irbesartan 300 mg (85%)^26^. These findings are consistent with our analytic trend, suggesting that higher doses of Azilsartan may be necessary to achieve optimal blood pressure control in some patients.

The high efficacy of Azilsartan compared to other drugs of this ARB can be explained by its mechanism of action. Azilsartan inhibits angiotensin II-induced vascular contractions and has an inverse agonism against AT1. The fact that one drug of the same class worked better than others could be explained and attributed to its “insurmountaiblity” or longer half-life, that is the formation of tight complexes that take longer to eliminate from the body, thus providing longer action^27,28^. This translates into producing significant and long-lasting antihypertensive effects. Hence, the observed reductions in SBP and DBP in our analysis further strengthen the position of Azilsartan as an effective therapeutic option for hypertensive patients, potentially contributing to improved patient outcomes and quality of life.

It is worth mentioning that there were fewer studies investigating the effects of lower doses, precisely 10 mg and 20 mg of Azilsartan^11,16,19^. Hence, we could not critically analyze them. This discrepancy in the available literature highlights an important area for future research. A more comprehensive understanding of the clinical outcomes associated with lower doses of Azilsartan could provide valuable insights into dose-dependent effects and help tailor treatment regimens to individual patient needs.

Introducing Azilsartan in regular practice may not only bring clinical benefits but can also be cost-effective. It is estimated that $93.5 billion per year are spent only to manage hypertension and its related disorders like stroke and cardiovascular events^29^. A one-month stock of azilsartan medoxomil at its highest potency (80 mg ) is more affordable compared to any other alternative ARBs currently present in the market. While other ARBs cost $113–$134, the maximum cost of Azilsartan rounds up to $90, thus making it an exceptionally budget-friendly option within its drug category^7^.

Like any other study, our paper has some limitations. Firstly, the restriction of data to four countries only highlights the need for more geographical variance for the study. Secondly, some studies did not report exact numbers, and eventually, data had to be extracted, which could have led to some numerical errors. Thirdly, many of the ARBs were not used as controls in our studies, highlighting the need for more trials of Azilsartan with every drug of its class. The clinical characteristics of our included study populations, including age, gender, baseline health status, and co-morbidities were variable, and the follow-ups of our studies were at different time points, leading to potential heterogeneity. Moreover, the side effect profile of each drug needed to be analyzed, paving the way for future research. We also observed a need for more data regarding lower Azilsartan doses, precisely 10 mg, and 20 mg, in the available literature. As a result, the findings for these lower doses are less robust. While our subgroup analysis highlighted the effectiveness of 40 mg and 80 mg of Azilsartan, it’s essential to consider variations in dosing regimens across different studies. Additionally, the studies included in this meta-analysis do not fully represent all patient populations or clinical scenarios. Moreover, while Azilsartan demonstrates better efficacy in our included population, two trials have reported it to have no superior benefit compared to other drugs of its class of diabetic patients. The drug had no effect on insulin resistance in hypertensive patients with concurring diabetes type II^15,17^. This warrants the need for trials, that focus on co-morbidities of the cohort, while analyzing the data. Lastly, our primary aim was to assess the clinical impact of Azilsartan in reducing BP in hypertensive patients within the context of routine clinical practice. We prioritized clinical SBP and DBP measurements taken under standard conditions over ambulatory BP as they directly relate to the management of hypertension.

## Conclusion

Our systematic review and meta-analysis highlight the significant clinical benefits of Azilsartan in reducing SBP and DBP. The observed trends in dose-dependent effects highlight the importance of considering the optimal dosing regimens when prescribing Azilsartan to hypertensive patients. These findings will prove valuable to clinicians for their everyday practice in managing hypertension effectively and will lay the groundwork for future research in this field.

## Ethical approval

Ethical approval was not required for conducting this systematic review study.

## Consent

Informed consent was not required for this systematic review study.

## Source of funding

No financial support was received for the conduct of this study.

## Author contribution

Q.A.K., I.U.H., and A.B. conceptualize the study. S.S., D.L., N.F.B., and A.M.F. did the literature search and extracted the data. Q.A.K. and M.A. analyzed the data, B.S., I.U.H., M.A. and A.M.F. wrote the original manuscript. A.B. and Q.A.K. critically revised and edited the final manuscript. All authors reviewed and approved the final manuscript before submission.

## Conflicts of interest disclosure

The authors declare that they have no conflicts of interest or financial interests related to the material of this manuscript.

## Research registration unique identifying number (UIN)

Registery used: PROSPERO Unique identifying number or registration ID: CRD42023456618 https://www.crd.york.ac.uk/prospero/#recordDetails.

## Guarantor

Qaisar Ali Khan.

## Data availability statement

Data can be available upon reasonable request to corresponding author.

## Provenance and peer review

Not commissioned, externally peer-reviewed.
